# Supplementary material for: Influence of road environmental factors on traffic accidents involving vulnerable road users through negative binomial models
Source: PLoS One. 2025 Feb 13;20(2):e0317601. doi: 10.1371/journal.pone.0317601 (PMC11824970; doi:10.1371/journal.pone.0317601)
Supplement: Attachment — Submitted filename: PONE-D-24-31325.pdf [file pone.0317601.s002.pdf]

PLOS ONE

Influence of Road Environmental Factors on Traffic Accidents Involving Vulnerable Road Users through Negative Binomial Models

--Manuscript Draft--

Manuscript Number:	PONE-D-24-31325
Article Type:	Research Article
Full Title:	Influence of Road Environmental Factors on Traffic Accidents Involving Vulnerable Road Users through Negative Binomial Models
Short Title:	Study on the Transportation Safety of Vulnerable Groups
Corresponding Author:	Yi Tian<br>Changsha University of Science and Technology<br>Changsha, Hunan Province CHINA
Keywords:	pedestrian safety; road environment; negative binomial models; transportation equity; vulnerable road users
Abstract:	Pedestrian safety is a critical public health and transportation equity concern, particularly in rapidly motorizing regions experiencing increasing vehicle-pedestrian conflicts. This study provides a comprehensive, data-driven analysis of the relationships between objectively measured road environment characteristics and pedestrian accident frequencies in Hunan Province, China. By leveraging detailed historical crash records linked to spatially-explicit infrastructure data, the research team employed advanced count regression modeling techniques, including negative binomial (NB) and zero truncated tail negative binomial (ZTNB) specifications, to systematically evaluate the safety impacts of roadway functional classification, intersection design, traffic controls, alignment geometry, pedestrian segregation, land use context, and traffic volumes. The results revealed that the ZTNB approach, which accounted for the excess zero observations inherent to the crash data, provided statistically superior model fit compared to the standard NB formulation. The ZTNB estimation results offered robust empirical evidence regarding key infrastructure risk factors, highlighting that while higher-order roadways exhibited lower pedestrian accident likelihoods, elements such as multi-leg intersections, lack of traffic controls, curved alignments, and absence of segregated facilities correlated with elevated hazards. Notably, the analysis also uncovered differential safety impacts across diverse user groups, including children and the elderly, emphasizing the importance of considering the distinct needs and vulnerabilities of various pedestrian populations in transportation planning and design. The findings underscore the critical importance of adopting a user-centric, age-sensitive approach to transportation infrastructure improvements, with targeted interventions focused on enhancing separation, controls, and accessibility tailored to the unique capacities of different pedestrian cohorts. Continued research exploring the linkages between the road environment, user behaviors, and collision severity can further elucidate comprehensive safety enhancement opportunities.
Order of Authors:	Ying Chen
	Yi Tian
	Zhaoheng Ouyang
	Jiaxun Zhu
Additional Information:	
Question	Response
Financial Disclosure<br><br>Enter a financial disclosure statement that describes the sources of funding for the work included in this submission. Review	Yes

Powered by Editorial Manager® and ProduXion Manager® from Aries Systems Corporation

the submission guidelines for detailed requirements. View published research articles from *PLOS ONE* for specific examples.<br><br>This statement is required for submission and **will appear in the published article** if the submission is accepted. Please make sure it is accurate.<br><br>**Funded studies**<br>Enter a statement with the following details:<br>• Initials of the authors who received each award<br>• Grant numbers awarded to each author<br>• The full name of each funder<br>• URL of each funder website<br>• Did the sponsors or funders play any role in the study design, data collection and analysis, decision to publish, or preparation of the manuscript?<br><br>Did you receive funding for this work?	
Please add funding details.<br>as follow-up to "**Financial Disclosure**<br><br>Enter a financial disclosure statement that describes the sources of funding for the work included in this submission. Review the submission guidelines for detailed requirements. View published research articles from *PLOS ONE* for specific examples.<br><br>This statement is required for submission and **will appear in the published article** if the submission is accepted. Please make sure it is accurate.	This work was supported in the form of grants from the National Natural Science Foundation of China(No.52102407) awarded to YC. The funders had no role in study design, data collection and analysis, decision to publish, or preparation of the manuscript.https://www.nsfc.gov.cn/

Powered by Editorial Manager® and ProduXion Manager® from Aries Systems Corporation

**Funded studies**<br>Enter a statement with the following details:<br>• Initials of the authors who received each award<br>• Grant numbers awarded to each author<br>• The full name of each funder<br>• URL of each funder website<br>• Did the sponsors or funders play any role in the study design, data collection and analysis, decision to publish, or preparation of the manuscript?<br><br>Did you receive funding for this work?"	
Please select the country of your main research funder (please select carefully as in some cases this is used in fee calculation).<br>as follow-up to "**Financial Disclosure**<br><br>Enter a financial disclosure statement that describes the sources of funding for the work included in this submission. Review the submission guidelines for detailed requirements. View published research articles from *PLOS ONE* for specific examples.<br><br>This statement is required for submission and **will appear in the published article** if the submission is accepted. Please make sure it is accurate.<br><br>**Funded studies**<br>Enter a statement with the following details:<br>• Initials of the authors who received each award<br>• Grant numbers awarded to each author<br>• The full name of each funder<br>• URL of each funder website<br>• Did the sponsors or funders play any role in the study design, data collection and analysis, decision to publish, or preparation of the manuscript?<br><br>Did you receive funding for this work?"	CHINA - CN
**Competing Interests**	The authors have declared that no competing interests exist.

Use the instructions below to enter a competing interest statement for this submission. On behalf of all authors, disclose any competing interests that could be perceived to bias this work—acknowledging all financial support and any other relevant financial or non-financial competing interests.<br><br>This statement is required for submission and **will appear in the published article** if the submission is accepted. Please make sure it is accurate and that any funding sources listed in your Funding Information later in the submission form are also declared in your Financial Disclosure statement.<br><br>View published research articles from *PLOS ONE* for specific examples.<br><br>**NO authors have competing interests**<br><br>Enter: *The authors have declared that no competing interests exist.*<br><br>**Authors with competing interests**<br><br>Enter competing interest details beginning with this statement:<br><br>*I have read the journal's policy and the authors of this manuscript have the following competing interests: [insert competing interests here]*<br><br>* typeset	
**Ethics Statement**<br><br>Enter an ethics statement for this submission. This statement is required if the study involved:<br><br>• Human participants<br>• Human specimens or tissue<br>• Vertebrate animals or cephalopods<br>• Vertebrate embryos or tissues	N/A

Powered by Editorial Manager® and ProduXion Manager® from Aries Systems Corporation

• Field research<br><br>Write "N/A" if the submission does not require an ethics statement.<br><br>General guidance is provided below. Consult the submission guidelines for detailed instructions. **Make sure that all information entered here is included in the Methods section of the manuscript.**	

**Format for specific study types**<br><br>**Human Subject Research (involving human participants and/or tissue)**<br>• Give the name of the institutional review board or ethics committee that approved the study<br>• Include the approval number and/or a statement indicating approval of this research<br>• Indicate the form of consent obtained (written/oral) or the reason that consent was not obtained (e.g. the data were analyzed anonymously)<br><br>**Animal Research (involving vertebrate animals, embryos or tissues)**<br>• Provide the name of the Institutional Animal Care and Use Committee (IACUC) or other relevant ethics board that reviewed the study protocol, and indicate whether they approved this research or granted a formal waiver of ethical approval<br>• Include an approval number if one was obtained<br>• If the study involved *non-human primates*, add *additional details* about animal welfare and steps taken to ameliorate suffering<br>• If anesthesia, euthanasia, or any kind of animal sacrifice is part of the study, include briefly which substances and/or methods were applied<br><br>**Field Research**<br><br>Include the following details if this study involves the collection of plant, animal, or other materials from a natural setting:<br>• Field permit number<br>• Name of the institution or relevant body that granted permission	
**Data Availability**<br><br>Authors are required to make all data underlying the findings described fully available, without restriction, and from the time of publication. PLOS allows rare exceptions to address legal and ethical concerns. See the PLOS Data Policy and FAQ for detailed information.	No - some restrictions will apply

Powered by Editorial Manager® and ProduXion Manager® from Aries Systems Corporation

A Data Availability Statement describing where the data can be found is required at submission. Your answers to this question constitute the Data Availability Statement and **will be published in the article**, if accepted.<br><br>**Important:** Stating 'data available on request from the author' is not sufficient. If your data are only available upon request, select 'No' for the first question and explain your exceptional situation in the text box.<br><br>Do the authors confirm that all data underlying the findings described in their manuscript are fully available without restriction?	
**Describe where the data may be found in full sentences. If you are copying our sample text, replace any instances of XXX with the appropriate details.**<br><br>• If the data are **held or will be held in a public repository**, include URLs, accession numbers or DOIs. If this information will only be available after acceptance, indicate this by ticking the box below. For example: *All XXX files are available from the XXX database (accession number(s) XXX, XXX.*).<br>• If the data are all contained **within the manuscript and/or Supporting Information files**, enter the following: *All relevant data are within the manuscript and its Supporting Information files.*<br>• If neither of these applies but you are able to provide **details of access elsewhere**, with or without limitations, please do so. For example:<br><br>*Data cannot be shared publicly because of [XXX]. Data are available from the XXX Institutional Data Access / Ethics Committee (contact via XXX) for researchers who meet the criteria for access to confidential data.*<br><br>*The data underlying the results presented in the study are available from (include the name of the third party*	Data cannot be shared publicly because of relevant laws and regulations in China.Data are available from Public Security Department, Hunan Province, Chinafor researchers who meet the criteria for access to confidential data.

and contact information or URL).<br>• This text is appropriate if the data are owned by a third party and authors do not have permission to share the data.<br><br>* typeset	
Additional data availability information:	

Manuscript
Click here to access/download;Manuscript;Influence of Road Environmental Factors.docx

Research Paper

Influence of Road Environmental Factors on Traffic Accidents Involving Vulnerable Road Users through Negative Binomial Models

Ying Chen[1], Yi Tian[2*], Zhaoheng Ouyang[3] and Jiaxun Zhu[4, #a]

[1]*College of Architecture, Changsha University of Science & Technology, Changsha Hunan Province China*

[2]*College of Architecture, Changsha University of Science & Technology, Changsha Hunan Province China*

[3]*College of Architecture, Changsha University of Science & Technology, Changsha Hunan Province China,*

[4]*College of Architecture, Changsha University of Science & Technology, Changsha Hunan Province China*

[#a]*current address: No. 45, Chiling Road, Changsha (Tianxin District), Hunan Province, China.*

**Corresponding author*

E-mail: mowangdal@163.com

Abstract: Pedestrian safety is a critical public health and transportation equity concern, particularly in rapidly motorizing regions experiencing increasing vehicle-pedestrian conflicts. This study provides a comprehensive, data-driven analysis of the relationships between objectively measured road environment characteristics and pedestrian accident frequencies in Hunan Province, China. By leveraging detailed historical crash records linked to spatially-explicit infrastructure data, the research team employed advanced count regression modeling techniques, including negative binomial (NB) and zero truncated tail negative binomial (ZTNB) specifications, to systematically evaluate the safety impacts of roadway functional classification, intersection design, traffic controls, alignment geometry, pedestrian segregation, land use context, and traffic volumes. The results revealed that the ZTNB approach, which accounted for the excess zero observations inherent to the crash data, provided statistically superior model fit compared to the standard NB formulation. The ZTNB estimation results offered robust empirical evidence regarding key infrastructure risk factors, highlighting that while higher-order roadways exhibited lower pedestrian accident likelihoods, elements such as multi-leg intersections, lack of traffic controls, curved alignments, and absence of

segregated facilities correlated with elevated hazards. Notably, the analysis also uncovered differential safety impacts across diverse user groups, including children and the elderly, emphasizing the importance of considering the distinct needs and vulnerabilities of various pedestrian populations in transportation planning and design. The findings underscore the critical importance of adopting a user-centric, age-sensitive approach to transportation infrastructure improvements, with targeted interventions focused on enhancing separation, controls, and accessibility tailored to the unique capacities of different pedestrian cohorts. Continued research exploring the linkages between the road environment, user behaviors, and collision severity can further elucidate comprehensive safety enhancement opportunitie[illegible]

Keywords: pedestrian safety; road environment; negative binomial models; transportation equity; vulnerable road users

1. Introduction

With the rapid expansion of China's highway network and continuous growth in motor vehicle ownership, the issues of chaotic traffic conditions and traffic accidents have remained persistent challenges. Road traffic injuries pose a significant burden globally, with profound health, social, and economic implications. It is estimated that one person dies in a traffic accident every 24 seconds worldwide [1]. Within this alarming statistic, China has experienced a disproportionately high number and percentage of pedestrian and occupant accidents compared to the total number of traffic crashes (Figure 1). Therefore, comprehending and addressing the factors contributing to pedestrian accidents, particularly among vulnerable populations, is vital from both an equity and public health standpoint.

Fig. 1 Number and share of pedestrian-occupant accidents in China's road traffic accidents, 2013-2021[2].

Specific demographic groups among pedestrians face a significantly elevated risk, notably children and older adults, who confront distinct safety challenges in complex traffic environments due to age-related physical and cognitive limitations. Road traffic injuries are the leading cause of death for children and young people aged 5−29 years [3]. For the elderly population, the mortality

rate in road crashes is alarmingly high [4], with the percentage of traffic accident fatalities among the elderly in China reaching 33.6% in 2018 [5]. Rates of pedestrian collisions are disproportionately high among children and older adults compared to the general population, underscoring their heightened vulnerability on roads. Existing research indicates that the physical road environment likely plays a pivotal, albeit intricate, role in determining accident risks for different user groups. Therefore, grappling with such a grave situation, understanding how to effectively mitigate the travel risks faced by these vulnerable groups has become a pressing concern. An essential aspect of this endeavor involves analyzing the role of the road environment in contributing to their traffic accidents [6].

To address these knowledge gaps, the objectives of this study were twofold: 1) to observe the distinct characteristics and differences between the two modeling approaches in capturing the relationship between road environment and traffic accidents, and 2) to derive insights into the travel patterns and safety issues faced by the elderly and young, thereby providing an evidence base to guide optimization of the road environment for improved protection of these vulnerable road users.

Through a comprehensive literature review, this introduction first established the context and rationale for focusing on the traffic safety of elderly and child pedestrians, highlighting the severity of the problem and the critical role of the road environment. It then outlined the specific research goals and modeling techniques employed in the current study. The subsequent sections delved deeper into the data sources, methodological approaches, analysis of results, and implications for enhancing traffic safety for vulnerable populations. Ultimately, this research aimed to contribute to the growing body of knowledge on the complex interplay between road infrastructure design and the safety of pedestrians, particularly those most at risk in the transportation system.

2. Literature Review

The existing literature has extensively examined the relationship between the road environment and pedestrian traffic safety. Several studies have revealed that changes in the road environment can cause changes in driving safety, changes involving pedestrians are the most hazardous and drivers perceive rural roads to be less hazardous than urban roads [7]. The design of roads and the development of different land uses have been shown to increase or decrease the road traffic injuries suffered by pedestrians [8]. Commercial or high-density residential areas concentrating pedestrian

activity tend to see higher crash frequencies, necessitating additional infrastructure supports, yet crash rates per travelled distance may be lower due to traffic calming from mixed usages. Conversely, sparse suburban or rural spacing of destinations discourages walking while facilitating high-speed motorized travel, negatively impacting pedestrian protection.

Researchers have identified various road environmental factors associated with pedestrian crash risk. Zewdie et al. [9] found that pedestrian crashes were linked to road environmental factors such as road density and traffic signals. Xu et al. [10] observed that higher traffic volume was associated with an increased likelihood of crashes, and there were differing associations between land use and crashes in urban and suburban areas. Gálvez-Pérez et al. [11] reported that wider sidewalks and higher signal densities were associated with reduced traffic accidents among elderly pedestrians. Amiour et al. [12] discovered that the presence of sidewalks was linked to the traffic safety of child pedestrians, and intersection density was related to children's safety perceptions. Aceves-González et al. [13] revealed that the absence of traffic signals, excessive traffic, and lack of signs were detrimental to pedestrian traffic safety. T Kraidi et al. [14] identified that both the number of cross-branching roads and cross-traffic volume were significant risk factors associated with pedestrian crash risk.

While effects of design on general pedestrian crash risks are reasonably well-established, research specifically targeting vulnerable elderly and child populations remains limited. Early studies on vulnerable groups, specifically the elderly and children, predominantly examined their behavior, environment, and other research starting points. Studies have revealed that in traffic accidents, the elderly are more likely to sustain injuries to the torso, pelvis, and limbs, with a lower incidence of head injuries, and exhibit a higher mortality rate in hospitals [15]. Ang et al. [16], in his investigation into the causes of death in road traffic accidents among the elderly, noted a higher mortality rate among pedestrians aged over 75 years. Ren et al. [17], in their controlled experiment comparing the mobility of the elderly with that of the young, determined that the extended time spent by the elderly at crossings was a primary factor contributing to their decreased mobility. Laković et al. [18], in their research, highlighted that suboptimal external conditions for older adults may expose them to more hazardous situations, potentially leading to misbehavior.

Regarding child pedestrians, studies have shown that less urbanized counties are associated with higher fatality rates in their examination of child fatalities from road traffic crashes in the

United States [19]. Chong et al. [20], in their research, highlighted that children are particularly susceptible to severe injuries when traveling as pedestrians. Wang et al. [21] identified that children's emotional fear could predict risk-taking behavior in traffic, with fear potentially leading to excessive caution and inefficiency in less risky traffic conditions. Zhao et al. [22] determined that the pedestrian accessibility and safety of children's school travel routes are influenced by factors such as effective walking width, spatial connectivity, visual integration, pedestrian safety obstacles, cross-facility completeness, and traffic flow.

More recent analyses have employed advanced statistical techniques to disentangle environmental influences from other determinants. Negative binomial (NB) regression allows controlling for confounding factors and exposure while detecting associations between attribute levels and injury counts. Zero truncated tail negative binomial (ZTNB) models further address issues of excess zeros in safety datasets and improve fitting for crash frequency analysis. By rigorously modeling the statistical properties of accident data, these analytical approaches can provide robust, data-driven evidence to guide targeted infrastructure interventions and policy decisions.

In summary, the extant literature highlights the versatility and power of count-based regression techniques, particularly the NB and ZTNB models, in uncovering the complex interplay between transportation infrastructure, land use, and the safety of vulnerable road users. However, the specific application of these methodologies to pedestrian safety issues in the Chinese context, with a focus on high-risk elderly and child populations, remains an underexplored area warranting further investigation. Therefore, this study aimed to address this gap by using historical traffic accident data from Hunan Province, China, to examine the relationship between the road environment and the traffic safety of the elderly and young groups.

3. Data and Methodology

3.1 Study Area and Data Sources

This study utilized traffic accident data collected by the Hunan Provincial Public Security Department in China, covering the 3-year period from 2014 to 2016. Hunan is a populous inland province located in south-central China, with a total area of around 210,000 square kilometers and a permanent residential population of over 65 million as of 2020. The provincial capital Changsha

and several other major cities in Hunan have experienced rapid urbanization and motorization in recent decades, leading to rising concerns over traffic safety, especially for vulnerable road users.

The accident dataset contained detailed information on each reported collision, including the date, time, location, vehicle types involved, and injury severity outcomes. For the current analysis, only records pertained to pedestrian accidents were extracted. The geocoded spatial coordinates provided in the dataset allowed linking each crash observation to corresponding roadway segments using GIS mapping software. This level of granularity in the data allows for a rigorous, quantitative examination of the influence of specific road environment factors on pedestrian accident frequencies.

Additionally, the age of persons involved in crashes was available, permitting stratification into vulnerable subgroups. Based on the data source the target population selected for this study is the elderly and children among the vulnerable groups on the road.Specifically, accidents involving pedestrians aged 60 years or above were categorized under the 'Elderly' group, while those relating to individuals between 1−12 years comprised the 'Child' group. All remaining observations not meeting these age criteria fell under a 'General' population category to serve as a baseline control group for comparative modeling.

After conducting data screening, which included missing data imputation and address geocoding, the final sample sizes comprised 1,682 accident records for the Elderly group, 659 records for Children, and 2,079 records representing the General pedestrians. Anomalous data can lead to misinterpretation when analyzing the results of fatal urban traffic accidents. Therefore, both the detection of abnormal data and the recovery of missing data are crucial in data preprocessing [23]. Traffic accident data from the entire year 2014−2016 in Hunan Province, China, underwent initial screening to replace missing values with the mean or median [24]. Due to the specificity of traffic accident statistics, the data was obtained from incidents with a number of accidents greater than zero (zero-truncated data). Subsequently, the accident locations were categorized based on road sections, and incidents occurring in the same road sections were aggregated. This process yielded a total of 1,682 groups of traffic accident data for the elderly, 659 groups for children, and 2,079 groups for non-children and elderly groups. The three data groups are structured as depicted in Figure 2.

Fig. 2 Comparison of the data structures of the three groups.

Utilizing statistically derived pedestrian traffic accident data, a heat map illustrating pedestrian traffic accidents in Hunan Province was generated through QGIS software (Figure 3). The map unveils the geospatial pattern of pedestrian traffic accidents, highlighting a concentration of incidents in urban areas and at intersections of road networks. This observational epidemiological methodology, leveraging actual crash exposure data, facilitates a direct evaluation of infrastructure safety across different populations, thereby surmounting constraints associated with self-reported metrics.

Fig. 3 Heat map of pedestrian traffic accidents in Hunan Province.

3.2 Environmental Variables

Based on prior literature substantiating their relevance to pedestrian safety and crash mechanisms, a set of roadway attributes reflecting urban form, traffic operations, and user separation characteristics were selected for systematic data collection and modeling. These factors were measured objectively along road segments corresponding to accident locations to characterize local infrastructural conditions at the time of each crash event.

Data on the accident location, road safety control, road alignment, intersection section type, road class, isolation type for classification and assignment of values were manually extracted from high-resolution satellite imagery and validated using online street view resources where available. Additionally, descriptive statistics of traffic volumes obtained from municipal count stations were joined spatially using GIS techniques.

Table 1 summarizes the environmental variables considered in the analysis and their coding schema, reflecting commonly adopted taxonomies in international road safety literature as well as China's national design guidelines. Composite safety control in road safety management refers to road control measures that incorporate two or more road control methods. Other safety facilities include lighting equipment, contour markers, guide signs, anti-glare installations, and additional safety measures. Special road sections within the intersection category refer to unique configurations such as tunnels, bridges, hazardous roadside areas, elevated structures, and roundabout intersections. Urban roads encompass first, second, third, and fourth class roads, while "others" denotes roads that do not meet the minimum standards of the lowest functional class of highways. Segregation Type 1 indicates the presence of a central barrier with a non-motorized

barrier, whereas Segregation Type 2 signifies the existence of either a central barrier or a non-motorized barrier alone.

Table 1 Road environment impact factors.

3.3 Modeling Approach

To quantify the relationships between the road environment characteristics and pedestrian accident frequencies, this study employed two distinct count-based regression modeling techniques: the NB model and the ZTNB model.

3.3.1 Negative binomial model

The NB model is a popular analytical tool in traffic safety [25]. The NB model is based on the prior Poisson prediction model, which is proposed when the Poisson prediction model is over- or under-discretized (fails to meet its fundamental assumptions that the mean and variance are equal). The NB model also follows the shape of the Poisson distribution, which is as follows:

$$P(Y|\mu) = \frac{\mu^{Y} \times e^{-\mu}}{Y!} \qquad Y = 0,1,2,\cdots \tag{1}$$

where Y represents the number of accidents occurring and μ the expected value of the number of accidents occurring, which can also be considered as a function that conforms to the conditions related to the impact of accidents, assuming that the mean value μ is related to the factors influencing the accidents.

$$\mu = f(\text{Road impact factors}) \tag{2}$$

When the data is taken to a non-zero value, the conditional probability in Eq. (1) is

$$P(Y|Y>0) = \frac{P(Y)}{P(Y>0)} = \frac{P(Y)}{1-P(Y=0)} \qquad Y = 0,1,2,\cdots \tag{3}$$

3.3.2 Zero truncated tail negative binomial models

Based on the NB model, Chen et al. [26,27] constructed the ZTNB model, which is used to better deal with the zero-truncated-tailed data containing only nonzero values. Assuming that μ obeys the Gamma distribution, α is the discrete coefficient in the Gamma distribution, the distribution probability function of the zero-truncated-tailed variable is finally obtained as:

$$P(Y=0|\mu,\alpha) = \frac{\Gamma\ (y+\alpha)}{\Gamma\ \ (Y+1)\times\Gamma(\alpha)} \times \left(\frac{\mu/\alpha}{1+(\frac{\mu}{\alpha})}\right)^{Y} \times \left(\frac{1}{1+(\frac{\mu}{\alpha})}\right)^{\alpha} = (\frac{1}{1+\frac{\mu}{\alpha}})^{\alpha} \tag{4}$$

$$P(Y|\mu,\alpha,Y>0) = \frac{\Gamma\ (y+\alpha)}{\Gamma\ (Y+1)\times\Gamma(\alpha)}\times\left(\frac{\mu/\alpha}{1+(\frac{\mu}{\alpha})}\right)^{Y}\times\left(\frac{1}{1+(\frac{\mu}{\alpha})}\right)^{\alpha}/(1-(\frac{1}{1+\frac{\mu}{\alpha}})^{\alpha}) \quad (5)$$

where Γ is the gamma distribution, Y is the number of vehicle accidents, and μ is the mean of the number of accidents.

In the NB model and the ZTNB model, after assuming that there is a relationship between the number of accidents and the remaining accident elements, the relationship between μ and the remaining accident elements is:

$$\log(\mu) = \beta_1 + \sum \beta_i \log(x_i) \quad (6)$$

where β_1 is the coefficient of the intercept, x_i is the assigned value of the various accident elements, and β_i is the expected corresponding coefficient. The solution of the NB model with ZTNB model is generally solved for the parameters using maximum likelihood estimation.

The key distinction is that the ZTNB model accounts for the fact that only non-zero accident counts are observed in the dataset, as opposed to the full distribution including zero values. This is achieved by incorporating an additional parameter that models the probability of a non-zero outcome, thereby providing more accurate estimates of the regression coefficients.

The ZTNB model is estimated using maximum likelihood techniques, with the log-likelihood function modified to account for the truncation at zero. The resulting coefficient estimates can be interpreted similarly to the NB model, representing the expected change in the log of the non-zero accident count associated with a unit change in the corresponding explanatory variable.

4. Comparison and Analysis of Results

4.1 Comparison of Results for the Elderly Group

The data structure and model results for the elderly group are presented in Tables 2 and 3. The NB model and ZTNB model both identified several significant factors related to the number of traffic accidents among the elderly.

Table 2 Data structure of the elderly group.

Table 3 Results of NB and ZTNB models for the elderly group.

The most influential factor was road class, with larger negative coefficients in both models (-0.1440 and -0.3286, respectively), indicating that as the road class increases (e.g., from lower-class urban roads to highways), the number of accidents involving the elderly tends to decrease. This

suggests that high-class roads may provide a safer environment for elderly pedestrians compared to lower-class urban roads. Other significant factors include accident location, road safety control, and intersection section type. The positive coefficient for accident location indicates that accidents are more likely to occur in more dangerous road sections, such as on the roadway rather than on sidewalks. The negative coefficients for road safety control and intersection section type suggest that better safety measures and less complex intersections are associated with fewer accidents involving the elderly. In contrast, road alignment and segregation type did not show significant relationships with accident frequency in the elderly group. This implies that factors like road curvature and the presence of physical barriers may not be the primary determinants of traffic safety for elderly pedestrians in this context.

4.2 Comparison of Results for the Children's Group

The data for the children's group were also analyzed using the NB and ZTNB models to investigate the relationship between road environment and traffic accident frequency. The data structure for the children's group and the model results are presented in Tables 4 and 5, respectively.

Table 4 Data structure of the Children's group.

Table 5 Results of NB and ZTNB models for the children's group.

In the two models for the children's group, significant factors influencing the number of traffic accidents included intersection section type and road grade. Notably, the estimated coefficient for road grade exhibited the most pronounced impact among the significant factors. Specifically, the estimated coefficients for road grade in both models were -0.1920 and -0.7381, respectively, indicating that road grade exerted the most substantial influence on the number of traffic accidents involving children at the same intersection section. Moreover, the analysis revealed a positive correlation between higher road grades and increased accident rates for children. Interestingly, the occurrence of accidents involving children was found to be lower in more specialized road sections. Conversely, other factors within the children’s group data, such as accident location, road safety control, road alignment, and segregation type, did not exhibit statistical significance in these two models.

4.3 Comparative Analysis of the Non-Children and Elderly Group Results

To determine the characteristics of pedestrian traffic accidents for the young and old groups, the non-children and elderly groups were used as a control group. Two models were fitted using the

data structure for the non-children and elderly groups (Table 6), and the results are presented in Table 7.

Table 6 Data structure of the non-children and elderly groups.

Table 7 Results of NB and ZTNB models for the non-children and elderly groups.

In both models for the non-children and elderly groups, road traffic control and road segregation were strongly associated with the number of traffic accidents. The type of segregation was the most significant factor, with estimated coefficients of -0.1384 and -0.2937 in the two models, respectively. This indicates that the more comprehensive the segregation, the fewer the locations where traffic accidents occurred. Additionally, the better the traffic regulation, the more sites where there were traffic accidents. Other characteristics, including accident location, intersection section type, road class, and road alignment, were not found to be significant in either model for the non-children and elderly groups.

The comparative analysis of the results across the different groups provides insights into the distinct relationships between road environment factors and traffic accident frequency for the elderly, children, and the non-children and elderly groups. These findings can inform the development of targeted interventions and the optimization of road environments to enhance the safety of vulnerable road users.

5. Discussion

5.1 Comparative Performance of Modeling Approaches

This study applied two count regression modeling techniques - the NB model and the ZTNB model - to examine the relationships between road environment characteristics and pedestrian accident frequencies among different groups in Hunan Province, China. A comparative analysis of the modeling results provides useful insights into the performance of these approaches.

In comparing the strengths and weaknesses of the models, the size of the Akaike Information Criterion (AIC) and Bayesian Information Criterion (BIC) values is commonly utilized, where smaller values indicate better predictive and fitting abilities of the models. As shown in Table 8, the AIC and BIC values of the ZTNB model are notably smaller than those of the NB model. Additionally, while the mean squared error (MSE) and root mean squared error (RMSE) were similar between the two approaches, the ZTNB specification still demonstrated marginally superior

predictive accuracy.

Table 8 Comparing the outcomes of the three groups of models.

Overall, the comparative assessment confirms that accounting for dataset properties through the ZTNB approach provides a more refined, accurate representation of complex safety phenomena compared to standard NB regression. This systematic comparison validates the zero-truncated model as the preferred analytical framework for the current study's intended purposes.

5.2 Key Findings and Vulnerable Group Insights

Beyond the advantages of the ZTNB specification demonstrated above, several substantive findings emerge from the quantitative analysis that provide useful insights for decision makers. Consistent with previous literature, infrastructure elements like functional classification, intersection design, traffic controls, geometry and segmentation were clearly associated with pedestrian accident likelihoods. However, certain attributes differed substantially in their effects depending on the vulnerable group.

The comparison of the parameter sizes for the three sets of influencing factors (Figure 4) provides additional insights:

Road grade is the most influential factor on the number of accidents in both young and elderly groups, however, the higher the road grade often means greater traffic flow and speed, the young and elderly groups of vehicle speed, trajectory judgment ability is weak, unable to grasp the complexity of the fast traffic scene. In the road environment, as the road level increases, clear and easy-to-understand traffic signs should be increased, while the accident-prone road in the case of the impact of driving safety, speed limits, speed bumps and other measures can be used appropriately to reduce the occurrence of traffic accidents.

Both young and elderly groups demonstrate that the number of accidents in more special junctions is lower, whereas the number of accidents in conventional crossroads is higher. First and foremost, the number of special junctions is lower than the number of regular crossroads, and unique intersections such as tunnels and bridges are easy to notice, increasing people's vigilance. This minimizes the likelihood of crossing unique road sections such as three-way junctions, resulting in fewer accidents [28]. Although the type of segregation does not show significant characteristics, some higher protection zones and barriers can be used to prevent pedestrians from entering or performing dangerous crossing behaviors on special road sections. Second, the bigger the number

of accidents on common routes, the more likely it is that the elderly and young are limited by their own reasons for lacking transportation safety awareness. Due to a lack of experience, children are frequently irresponsible and hasty, and they lack basic transportation awareness [29]. Elderlyer pedestrians who use traditional mobility aids such as canes, walkers, and wheelchairs on a daily basis are more likely to be involved in pedestrian accidents [30]. In some areas where there are large groups of young and elderly group, traffic signals can be adjusted to allow enough time for them to pass, or in the roadway to reduce the number of accidents involving young and elderly groups by increasing the number of traffic police officers directing the roadway and guiding the roadway by a special person at the intersection.

Accident location is a road element that primarily impacts elderlyer age groups. Accidents are more common on dangerous roads, such as motorways and motorized/non-motorized roads. The primary cause for this is the limitations of elderly people's own variables and their reduced capacity to regulate traffic situations. Younger pedestrians cross the street more safely because they pay closer attention to motor traffic, but senior pedestrians pay more attention to the street since they must plan their steps better [31]. When crossing the street, elderly pedestrians turn their heads less frequently, and they estimate the distance to approaching automobiles less accurately [32]. It is probable that as people age, their judgment of road conditions deteriorates, and they fail to comply with traffic restrictions, putting them in unsafe situations where accidents are common. Separation zones and protection zones on roadways are still effective strategies to keep the elderly out of dangerous locations, and the proper placement of pedestrian islands in the middle of the road will make it much easier for the slow-moving elderly to cross. Where there is a greater need to cross, pedestrian crossings, bridges, or underpasses can be built to separate the elderly from automobiles and lessen the likelihood of an accident.

In terms of traffic control, the elderly and non-elderly populations are more likely to be engaged in many accidents in areas with superior traffic management, such as those with traffic lights, signs, and markings. In areas with good traffic equipment, the appearance of some violations may have a negative impact, which may be age related. Elderly persons often disregard the right of way, and poor vehicle movement prediction can contribute to accidents [33]. Young people, unlike the elderly, use technological devices to divert themselves [34]. This is done in accident-prone areas by employing signals to enhance pedestrian passage time without influencing traffic congestion,

changing the type of traffic control, such as adding manual traffic control, and more accident-prone signage to encourage vehicles to slow down.

In the case of the barrier, its existence makes it impossible for vehicles to move outside the regular travel area, and violations such as crossing the barrier by non-elderly or young individuals enhance the likelihood of an accident. In the parameters used, there is no significant relationship between road linearity and traffic accidents. In terms of road linearity, the presence of curves, up and down slopes reduce the speed of the vehicle due to the driver's initiative, and in special road linearity, people concentrate their attention, reducing the likelihood of accidents, contrary to our subconscious belief that curves and slopes have a very strong relationship with the occurrence of accidents.

Fig. 4. Comparison of parameter sizes for three sets of affecting factors.

Further analysis of the data revealed that the positive and negative signs of the estimated coefficients of the two model comparisons within the same group were consistent, but the magnitude of the values varied, with the estimated coefficients in the ZTNB model all being larger, indicating a greater influence on the dependent variable. In the comparison of the same models in three groups, the positive and negative sign of the significant factors did not change, indicating that there is indeed a potentially influential relationship between the influencing factors and the number of accidents in the accident data, and that the magnitude and significance of the influences are not the same in the different models.

5.3 Limitations and Future Research

Several limitations remain that future work could help address. First, unobserved confounding by unmeasured risk factors possibly associated with the infrastructure characteristics could bias results to an unknown extent. However, controlling for a large number of road environment covariates aimed to minimize this threat to internal validity.

Second, the generalizability of insights beyond the specific study context of Hunan Province, China, with unique infrastructure standards and travel patterns, requires cautious interpretation and validation elsewhere. Nevertheless, enhancing road safety science globally relies on evidence accretion from diverse transportation settings.

Third, the cross-sectional study design precludes conclusions regarding causal directionality

between variables. Extending models longitudinally could help establish temporality and monitor safety impacts of infrastructure modifications over time. Additionally, jointly modeling crash frequencies and severity outcomes may further disentangle risk factor relationships.

In summary, addressing limitations through expanded datasets, comparative analyses, theoretical model enhancements, and rigorous evaluation of countermeasure implementation will support continually progressing towards comprehensive, data-driven transportation safety solutions attuned to diverse user needs. The current study advances this objective for protecting vulnerable populations through a critical systems-level lens.

6. Conclusions

This study employed rigorous count-based regression modeling techniques to disentangle the complex relationship between the road environment and traffic accident risks faced by vulnerable road user groups, including the elderly and children, in China's Hunan Province. The application of both NB and ZTNB models allowed for robust examination of the factors influencing pedestrian accident frequencies while accounting for the unique statistical properties of the accident data.

The analysis revealed distinct patterns in how the road environment shapes safety outcomes for the elderly versus children. For the elderly group, key influential factors included road class, accident location, road safety control measures, and intersection configurations. Specifically, the results indicated that higher-class roads, improved safety infrastructure, and simpler intersections were associated with lower accident frequencies among elderly pedestrians. In contrast, the child pedestrian group was found to be more sensitive to road grade, with steeper gradients significantly increasing their crash risk. Intersection complexity also emerged as a notable determinant for child traffic safety.

These divergent findings underscore the need for tailored, age-segmented approaches to transportation planning and infrastructure design. Measures to enhance the mobility and safety of the elderly, such as expanding accessibility on urban streets, upgrading crossing facilities, and implementing traffic calming features, may be crucial. For child pedestrians, strategies focused on reducing speed differentials, improving crossing opportunities, and strengthening separation between motorized and non-motorized modes could yield substantial safety benefits.

By providing robust, data-driven evidence on the nuanced relationships between the road

environment and the safety of vulnerable road users, this study contributes to the growing literature on transportation equity and sustainable mobility. The insights gained can inform the development of more inclusive and age-friendly transportation systems, ultimately advancing the goal of Vision Zero and ensuring safe, accessible journeys for all members of the community.

Data Availability: All the relevant data are within the paper and its Supporting Information files.

Funding: This work was supported in the form of grants from *the National Natural Science Foundation of China*(No.52102407) awarded to YC. The funders had no role in study design, data collection and analysis, decision to publish, or preparation of the manuscript.

Competing interests: The authors have declared that no competing interests exist.

References

1. One person is killed in a traffic accident every 24 seconds UN calls for safer roads (un.org). United Nations. 2021; 11:21. Available from: https://news.un.org/zh/story/2021/11/1094772
2. National data. Death toll in traffic accidents;2023[cited 2024 July 10]; Available from: https://data.stats.gov.cn/easyquery.htm?cn=C01&zb=A0S0D01&sj=2022
3. Road traffic injuries.World Health Organization.2023;12:13. Available from:https://www.who.int/news-room/fact-sheets/detail/road-traffic-injuries.
4. Etehad H, Yousefzadeh-Chabok S, Davoudi-Kiakalaye A, Dehnadi AM, Hemati H, Mohtasham-Amiri Z. Impact of road traffic accidents on the elderly. Archives of Gerontology and Geriatrics. 2015;61(3):489-93. doi: 10.1016/j.archger.2015.08.008. PubMed PMID: WOS:000368012900025.
5. Traffic Administration of Ministry of Public Security . Annual Report on Road Traffic Accidents of the People Republic of China(2016—2018)[R] . Beijing:Traffic Administration of the Ministry of Public Security，2016－2018 .
6. Theeuwes J. Self-explaining roads: What does visual cognition tell us about designing safer

roads? Cognitive Research-Principles and Implications. 2021;6(1). doi: 10.1186/s41235-021-00281-6. PubMed PMID: WOS:000625541900001.

7. Cox JA, Beanland V, Filtness AJ. Risk and safety perception on urban and rural roads: Effects of environmental features, driver age and risk sensitivity. Traffic Injury Prevention. 2017;18(7):703-10. doi: 10.1080/15389588.2017.1296956. PubMed PMID: WOS:000407255900005.

8. Stoker P, Garfinkel-Castro A, Khayesi M, Odero W, Mwangi MN, Peden M, et al. Pedestrian Safety and the Built Environment: A Review of the Risk Factors. Journal of Planning Literature. 2015;30(4):377-92. doi: 10.1177/0885412215595438. PubMed PMID: WOS:000363899800002.

9. Zewdie HY, Sarmiento OL, Pinzón JD, Wilches-Mogollon MA, Arbelaez PA, Baldovino-Chiquillo L, et al. Road Traffic Injuries and the Built Environment in Bogotá, Colombia, 2015-2019: A Cross-Sectional Analysis. Journal of Urban Health-Bulletin of the New York Academy of Medicine. 2024. doi: 10.1007/s11524-024-00842-2. PubMed PMID: WOS:001198505400001.

10. Xu C, Zhang ZM, Fu FJ, Yao WB, Su HY, Hu YW, et al. Analysis of Spatiotemporal Factors Affecting Traffic Safety Based on Multisource Data Fusion. Journal of Transportation Engineering Part a-Systems. 2023;149(10). doi: 10.1061/jtepbs.Teeng-7990. PubMed PMID: WOS:001048431400010.

11. Gálvez-Pérez D, Guirao B, Ortuño A, Picado-Santos L. The Influence of Built Environment Factors on Elderly Pedestrian Road Safety in Cities: The Experience of Madrid. International Journal of Environmental Research and Public Health. 2022;19(4). doi: 10.3390/ijerph19042280. PubMed PMID: WOS:000769006000001.

12. Amiour Y, Waygood EOD, van den Berg PEW. Objective and Perceived Traffic Safety for Children: A Systematic Literature Review of Traffic and Built Environment Characteristics Related to Safe Travel. International Journal of Environmental Research and Public Health. 2022;19(5). doi: 10.3390/ijerph19052641. PubMed PMID: WOS:000768504100001.

13. Aceves-González C, Ekambaram K, Rey-Galindo J, Rizo-Corona L. The role of perceived pedestrian safety on designing safer built environments. Traffic Injury Prevention. 2020;21:S84-S9. doi: 10.1080/15389588.2020.1812062. PubMed PMID:

WOS:000569797700001.

14. Kraidi R, Evdorides H. Pedestrian safety models for urban environments with high roadside activities. Safety Science. 2020;130. doi: 10.1016/j.ssci.2020.104847. PubMed PMID: WOS:000560700900009.
15. Sadeghi-Bazargani H, Samadirad B, Moslemi F. A decade of road traffic fatalities among the elderly in north-West Iran. Bmc Public Health. 2018;18. doi: 10.1186/s12889-017-4976-2. PubMed PMID: WOS:000419646700001.
16. Ang BH, Chen WS, Lee SWH. Global burden of road traffic accidents in older adults: A systematic review and meta-regression analysis. Archives of Gerontology and Geriatrics. 2017; 72:32-8. doi: 10.1016/j.archger.2017.05.004. PubMed PMID: WOS:000408022200006.
17. Ren XX, Zhang J, Hu YH, Cao SC, Yang XX, Song WG. Spatial and temporal analysis of bottleneck flow for the elderly and the young pedestrians under multiple modes. Physica a-Statistical Mechanics and Its Applications. 2024;640. doi: 10.1016/j.physa.2024.129722. PubMed PMID: WOS:001216402000001.
18. Lakovic S, Tollazzi T, Gruden C. Elderly Pedestrians and Road Safety: Findings from the Slovenian Accident Database and Measures for Improving Their Safety. Sustainability. 2023;15(2). doi: 10.3390/su15021631. PubMed PMID: WOS:000927681700001.
19. Mokdad AA, Wolf LL, Pandya S, Ryan M, Qureshi FG. Road Traffic Accidents and Disparities in Child Mortality. Pediatrics. 2020;146(5). doi: 10.1542/peds.2019-3009. PubMed PMID: WOS:000589620500038.
20. Chong SL, Tyebally A, Chew SY, Lim YC, Feng XY, Chin ST, et al. Road traffic injuries among children and adolescents in Singapore - Who is at greatest risk? Accident Analysis and Prevention. 2017; 100:59-64. doi: 10.1016/j.aap.2017.01.005. PubMed PMID: WOS:000394557600008.
21. Wang HR, Morgan C, Li DQ, Huang R, Schwebel DC. Children's fear in traffic and its association with pedestrian decisions. Journal of Safety Research. 2021; 76:56-63. doi: 10.1016/j.jsr.2020.11.010. PubMed PMID: WOS:000641968500007.
22. Zhao J, Su W, Luo JC, Zuo J. Evaluation and Optimization of Walkability of Children's School Travel Road for Accessibility and Safety Improvement. International Journal of Environmental Research and Public Health. 2022;19(1). doi: 10.3390/ijerph19010071. PubMed PMID:

WOS:000751051500001.

23. Zhang X, Qi SM, Zheng A, Luo Y, Hao SQ. Data-Driven Analysis of Fatal Urban Traffic Accident Characteristics and Safety Enhancement Research. Sustainability. 2023;15(4). doi: 10.3390/su15043259. PubMed PMID: WOS:000942070500001.
24. Shen YJ, Hermans E, Bao Q, Brijs T, Wets G. Towards better road safety management: Lessons learned from inter-national benchmarking. Accident Analysis and Prevention. 2020;138. doi: 10.1016/j.aap.2020.105484. PubMed PMID: WOS:000537525400017.
25. Mao HY, Deng XW, Lord D, Flintsch G, Guo F. Adjusting finite sample bias in traffic safety modeling. Accident Analysis and Prevention. 2019; 131:112-21. doi: 10.1016/j.aap.2019.05.026. PubMed PMID: WOS:000484871700011.
26. Chen Y, Huang ZX. Predicting Wet-Road Crashes Using the Finite-Mixture Zero-Truncated Negative Binomial Model. Journal of Advanced Transportation. 2020;2020. doi: 10.1155/2020/8828939. PubMed PMID: WOS:000591563400001.
27. Chen Y, Yuan HZ, Huang ZX, Wang L, et al. Modeling Intersection Traffic Crashes Using a Zero-truncated Negative Binomial Model. China Journal of Highway and Transport.2020;33(04): 146-154.DOI: 10.19721/j.cnki.1001-7372.2020.04.015.
28. Kim D. The transportation safety of elderly pedestrians: Modeling contributing factors to elderly pedestrian collisions. Accident Analysis and Prevention. 2019; 131:268-74. doi: 10.1016/j.aap.2019.07.009. PubMed PMID: WOS:000484871700027.
29. Trifunovic A, Pesic D, Cicevic S, Antic B. The importance of spatial orientation and knowledge of traffic signs for children's traffic safety. Accident Analysis and Prevention. 2017; 102:81-92. doi: 10.1016/j.aap.2017.02.019. PubMed PMID: WOS:000401042800009.
30. Doulabi S, Hassan HM, Ferguson MR, Razavi S, Paez A. Exploring the determinants of older adults' susceptibility to pedestrians' incidents. Accident Analysis and Prevention. 2021;155. doi: 10.1016/j.aap.2021.106100. PubMed PMID: WOS:000644833800003.
31. Zito GA, Cazzoli D, Scheffler L, Jäger M, Müri RM, Mosimann UP, et al. Street crossing behavior in younger and older pedestrians: an eye- and head-tracking study. Bmc Geriatrics. 2015;15. doi: 10.1186/s12877-015-0175-0. PubMed PMID: WOS:000367368300002.
32. Choi J, Tay R, Kim S, Jeong S. Behaviors of older pedestrians at crosswalks in South Korea. Accident Analysis and Prevention. 2019; 127:231-5. doi: 10.1016/j.aap.2019.03.005. PubMed

PMID: WOS:000466830200024.

33. Lakovic S, Tollazzi T, Gruden C. Elderly Pedestrians and Road Safety: Findings from the Slovenian Accident Database and Measures for Improving Their Safety. Sustainability. 2023;15(2). doi: 10.3390/su15021631. PubMed PMID: WOS:000927681700001.

34. Escobar DA, Cardona S, Hernández-Pulgarín G. Risky pedestrian behaviour and its relationship with road infrastructure and age group: An observational analysis. Safety Science. 2021;143. doi: 10.1016/j.ssci.2021.105418. PubMed PMID: WOS:000690367600017.

Figure1

Click here to access/download;Figure;Fig1.tif

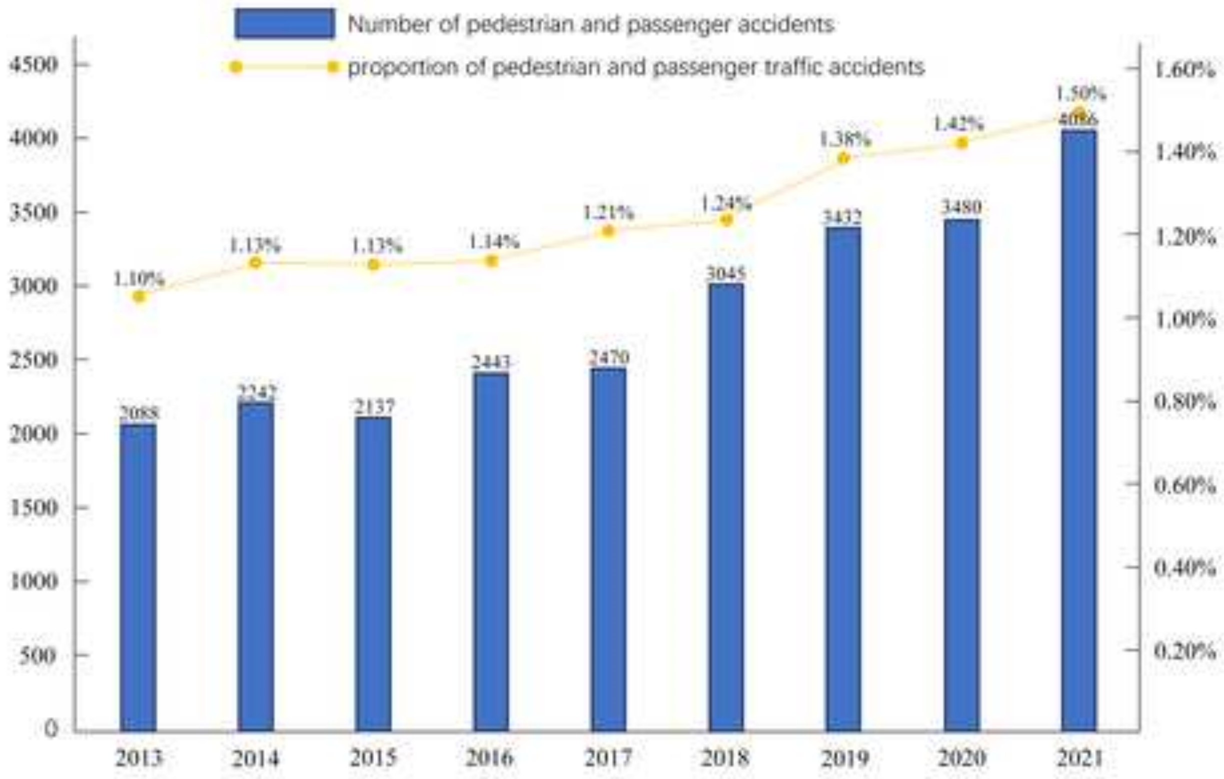

Figure2

Click here to access/download;Figure;Fig2.tif

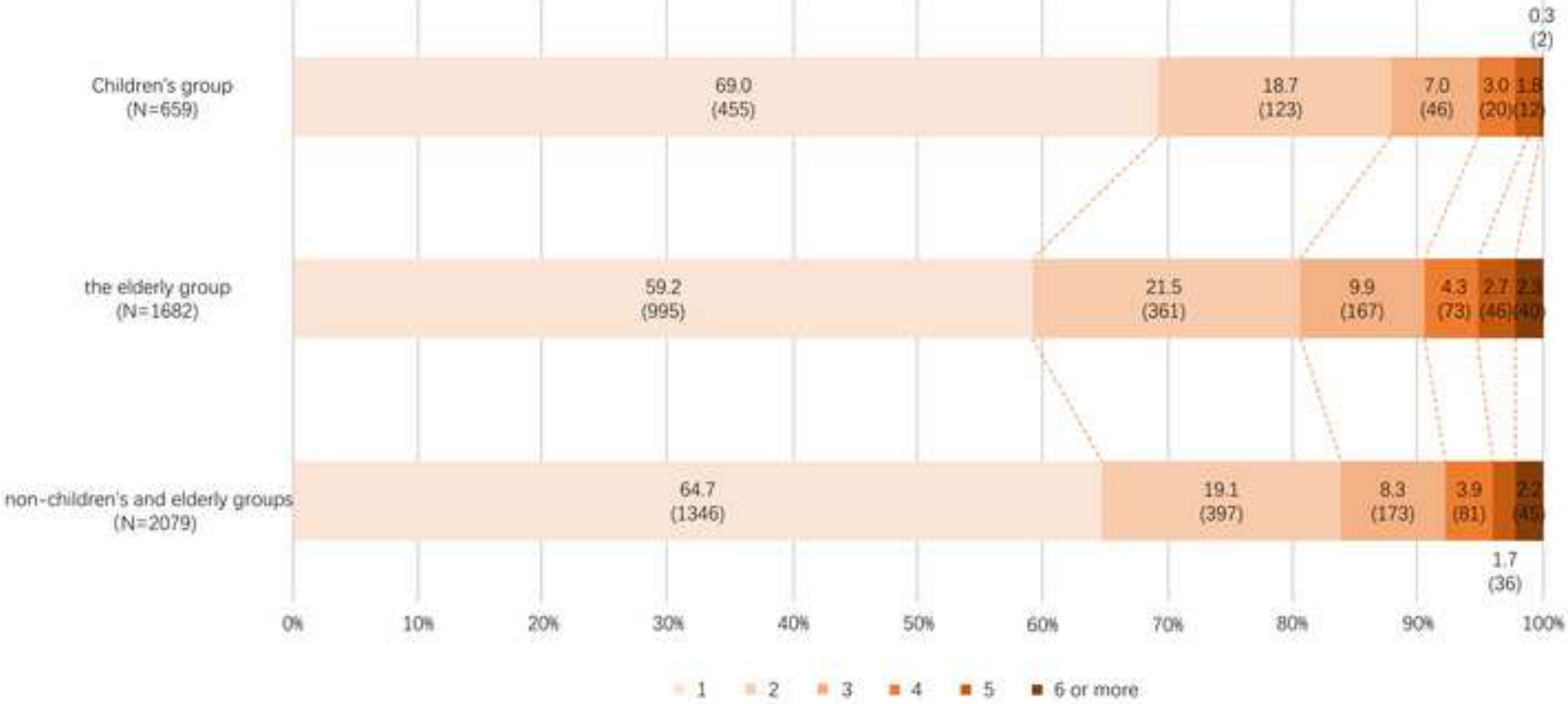

Figure3

Click here to access/download;Figure;Fig3.tif

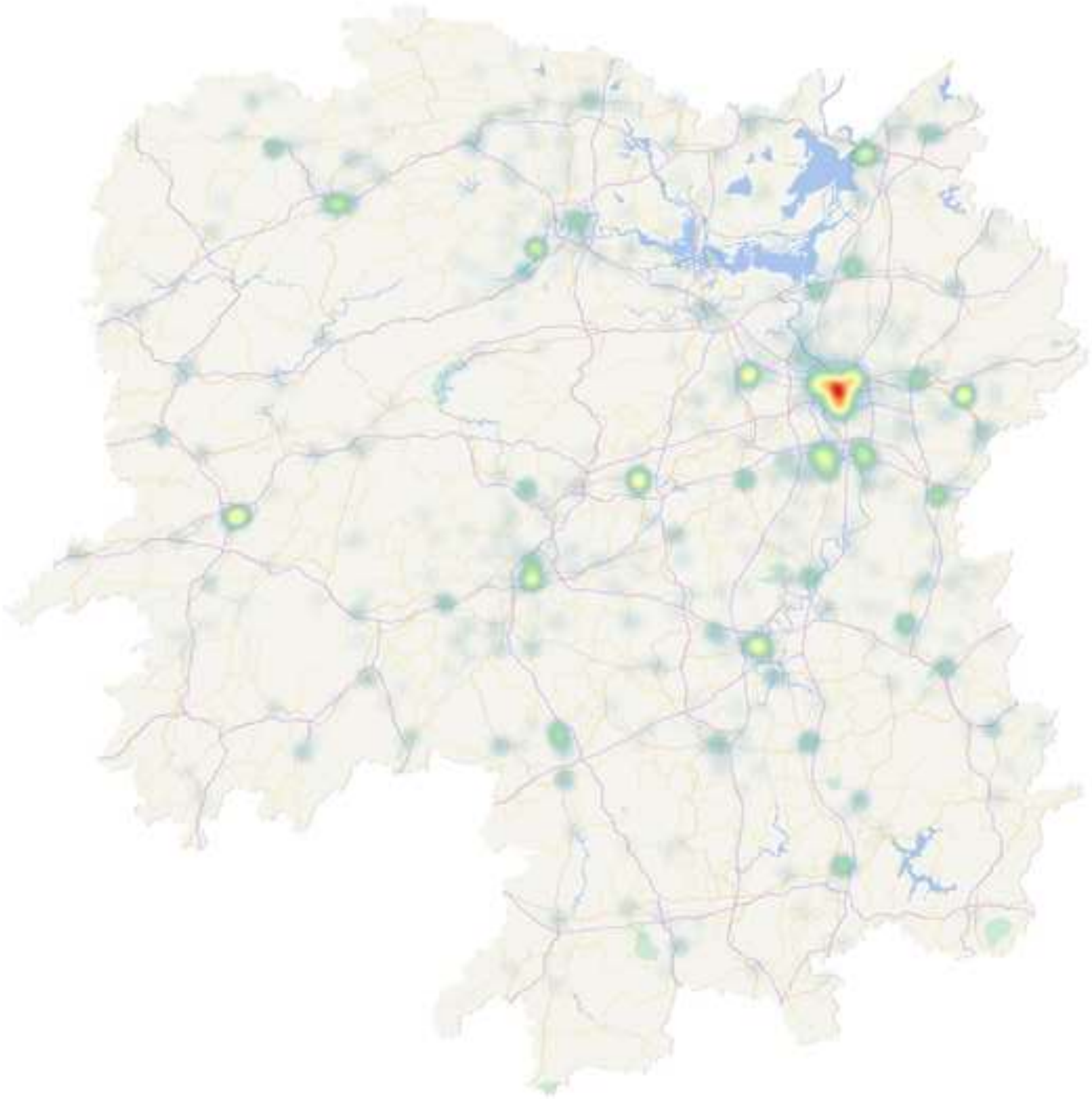

Figure4

Click here to access/download;Figure;Fig4.tif

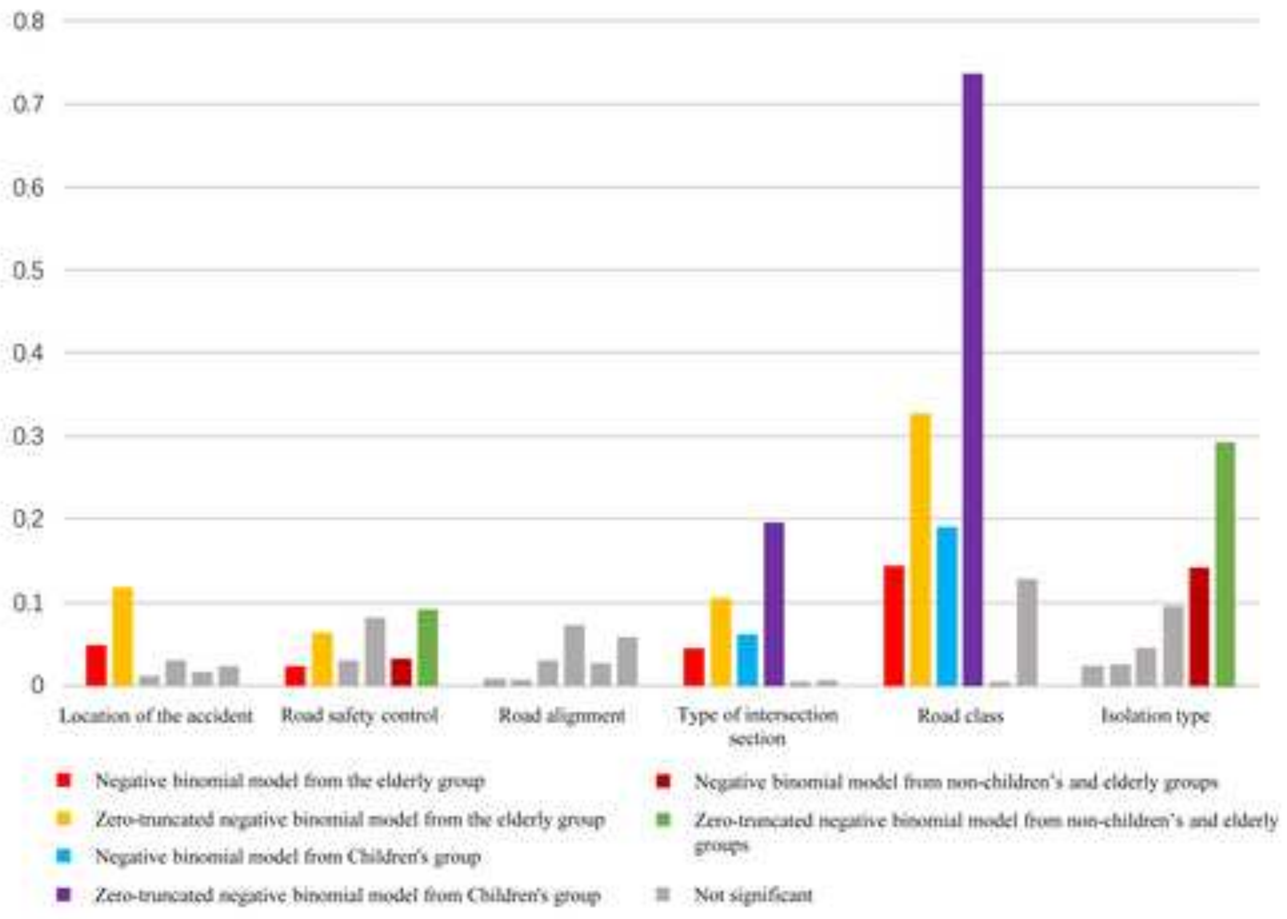

Impact Factors	Description	Value Assignment
Location of the accident	Sidewalks	1
	Pedestrian crossing	2
	Nonmotorized road	3
	Nonmotorized Mixed Lane	4
	motorway	5
Road safety control	Composite safety control	1
	Car indicator	2
	Other security facilities	3
	Traffic marking	4
	Traffic sign	5
	Uncontrolled	6
Road alignment	Straight	1
	Winding road	2
	Ramp	3
	Winding slope	4
Type of intersection section	Ordinary road	1
	Narrow road	2
	Access to the roadway	3
	Branch port	4
	Other special road sections	5
Road class	Freeway	1
	City road	2
	Other roads	3
Isolation type	Segregation Type 1	1
	Segregation Type 2	2
	Unisolated	3

 Click here to access/download;Figure;table2.tif

Parameter	Maximum Values	Minimum Value	Average Value
Number of accidents	6.000	1.000	1.772
Location of the accident	5.000	1.000	4.357
Road safety control	6.000	1.000	3.891
Road alignment	4.000	1.000	1.424
Type of intersection section	5.000	1.000	1.887
Road class	3.000	1.000	2.096
Isolation type	3.000	1.000	2.770

 Click here to access/download;Figure;table3.tif

Parameter	NB Model				ZTNB Model			
	Estimated	Standard deviation	P-value	Significance	Estimated	Standard deviation	P-value	Significance
β_1	0.7898	0.1619	1.07e-06	99.9%	0.1044	0.3703	0.7779	Not significant
β_2	——	——	——	——	-0.3563	0.2634	0.1760	Not significant
β_{LOC}	0.0495	0.0186	0.0079	99.0%	0.1184	0.0408	0.0037	99.0%
β_{CON}	-0.0263	0.0093	0.0047	99.0%	-0.0642	0.0201	0.0013	99.0%
β_{ALI}	-0.0066	0.0230	0.7712	Not significant	-0.0074	0.0495	0.8802	Not significant
β_{INT}	-0.0444	0.0133	0.0008	99.9%	-0.1059	0.0289	0.0002	99.9%
β_{ROA}	-0.1440	0.0497	0.0037	99.0%	-0.3286	0.1082	0.0023	99.0%
β_{LEO}	0.0206	0.0434	0.6337	Not significant	0.0256	0.0929	0.7824	Not significant
AIC	5056.16	——	——	——	4042.12	——	——	——
BIC	5099.58	——	——	——	4085.54	——	——	——
MSE	1.3947	——	——	——	1.3943	——	——	——
RMSE	1.1810	——	——	——	1.1808	——	——	——

 Click here to access/download;Figure;table4.tif

Parameter	Maximum Values	Minimum Value	Average Value
Number of accidents	8.000	1.000	1.516
Location of the accident	5.000	1.000	4.332
Road safety control	6.000	1.000	4.930
road alignment	4.000	1.000	1.458
Type of intersection section	5.000	1.000	1.624
road class	4.000	1.000	2.164
Isolation type	3.000	1.000	2.906

table5 Click here to access/download;Figure;table5.tif

Parameter	NB Model				ZTNB Model			
	Estimated	Standard deviation	P-value	Significance	Estimated	Standard deviation	P-value	Significance
β_1	0.8488	0.3394	0.0124	95.0%	0.5954	0.9128	0.5142	Not significant
β_2	——	——	——	——	-0.8742	0.7186	0.2238	Not significant
β_{LOC}	0.0129	0.0291	0.6561	Not significant	0.0308	0.0700	0.6598	Not significant
β_{COM}	-0.0317	0.0188	0.0923	Not significant	-0.0840	0.0444	0.0586	Not significant
β_{ALI}	0.0296	0.0410	0.4708	Not significant	0.0735	0.0979	0.4527	Not significant
β_{INT}	-0.0620	0.0269	0.0213	95.0%	-0.1975	0.0676	0.0035	99.0%
β_{ROA}	-0.1920	0.0883	0.0298	95.0%	-0.7381	0.2278	0.0012	99.0%
β_{JEO}	0.0457	0.1017	0.6533	Not significant	0.0976	0.2372	0.6806	Not significant
AIC	1792.57	——	——	——	1264.07	——	——	——
BIC	1828.49	——	——	——	1299.99	——	——	——
MSE	0.8781	——	——	——	0.8781	——	——	——
RMSE	0.9371	——	——	——	0.9365	——	——	——

Parameter	Maximum Values	Minimum Value	Average Value
Number of accidents	9.000	1.000	1.669
Location of the accident	5.000	1.000	4.574
Road safety control	6.000	1.000	4.930
Road alignment	4.000	1.000	1.363
Type of intersection section	5.000	1.000	1.846
Road class	4.000	1.000	2.047
Isolation type	3.000	1.000	2.734

Parameter	NB Model				ZTNB Model			
	Estimated	Standard deviation	P-value	Significance	Estimated	Standard deviation	P-value	Significance
β_1	1.1358	0.1574	5.49e-13	99.9%	-0.1548	0.7306	0.8321	Not significant
β_2	——	——	——	——	-2.0233	0.7478	0.0068	99.0%
β_{LOC}	-0.0169	0.0207	0.4141	Not significant	-0.0257	0.0541	0.6345	Not significant
β_{CON}	-0.0334	0.0085	8.86e-05	99.9%	-0.0933	0.0218	1.94e-05	99.9%
β_{ALI}	-0.0270	0.0244	0.2679	Not significant	-0.0580	0.0622	0.3507	Not significant
β_{INT}	-0.0042	0.012	0.7246	Not significant	-0.0070	0.0310	0.8212	Not significant
β_{ROA}	-0.0045	0.0443	0.9189	Not significant	-0.1301	0.1151	0.2582	Not significant
β_{ISO}	-0.1384	0.0362	0.0001	99.9%	-0.2937	0.0957	0.0021	99.0%
AIC	6111.97	——	——	——	4582.87	——	——	——
BIC	6157.09	——	——	——	4627.98	——	——	——
MSE	1.40	——	——	——	1.40	——	——	——
RMSE	1.18	——	——	——	1.18	——	——	——

table8 Click here to access/download;Figure;table8.tiff

	Children's Group		Elderly Group		non-children and elderly groups	
	NB model	ZTNB model	NB model	ZTNB model	NB model	ZTNB model
Location of the accident	Not significant	Not significant	99.0%	99.0%	Not significant	Not significant
Road safety control	Not significant	Not significant	99.0%	99.0%	99.9%	99.9%
Type of intersection section	95.0%	95.0%	99.9%	99.9%	Not significant	Not significant
Road class	95.0%	95.0%	99.0%	99.0%	Not significant	Not significant
Isolation type	Not significant	Not significant	Not significant	Not significant	99.9%	99.0%
Aic	1792.57	1264.07	5056.16	4042.12	6111.97	4582.87
Bic	1828.49	1299.99	5099.58	4085.54	6157.09	4627.98
Mse	0.87	0.87	1.39	1.39	1.40	1.40
Rmse	0.93	0.93	1.18	1.18	1.18	1.18

data

Click here to access/download

Supporting Information - Compressed/ZIP File Archive

data.zip
