# Supplementary material for: Influence of road environmental factors on traffic accidents involving vulnerable road users through negative binomial models
Source: PLoS One. 2025 Feb 13;20(2):e0317601. doi: 10.1371/journal.pone.0317601 (PMC11824970; doi:10.1371/journal.pone.0317601)
Supplement: Attachment — Submitted filename: reviewer decision.pdf [file pone.0317601.s004.pdf]

After extensive review of the research with the symbol ModelsPONE-D-24-31325R1and tagged Influence of Road Environmental Factors on Traffic Accidents Involving Vulnerable Road Users through Negative Binomial, the following was reached:

The reviewer recommends accepting the aforementioned research after observing the following:

1. The researcher made all the modifications required by him and determined by the four evaluators.
2. The researcher answered most of the research inquiries directed by reviewers.
3. Although the study period was proposed, which is the years 2014 to 2016, and although the years are not specific, the analysis was done in good ways and the study can be used for good future research.

With respect.

Asst.Prof.Dr.Rana Amir Yousif
