# Supplementary material for: Influence of road environmental factors on traffic accidents involving vulnerable road users through negative binomial models
Source: PLoS One. 2025 Feb 13;20(2):e0317601. doi: 10.1371/journal.pone.0317601 (PMC11824970; doi:10.1371/journal.pone.0317601)
Supplement: Attachment — Submitted filename: Response to Reviewers.docx [file pone.0317601.s003.docx]

**Author's Response to Reviewer’s Comments**

Dear Editors and Reviewers:

Thank you for your efficient work in processing our manuscript (PONE-D-24-31325). Those comments are very beneficial to improve our paper, and they also can be seen as the important guidance to our research. We have studied comments carefully and have made targeted revisions. The changes are marked red in the revised manuscript. Point by point responses to the reviewers’ comments are listed below.

In accordance with the laws and regulations stipulated by the pertinent authorities in China, the confidentiality and privacy of traffic accident data must be upheld and cannot be divulged.

**Reviewer 1**

**1. The abstract need more clear about the main finding and conclusions of the research.**

**Response:** Thank you for your feedback. We have revised the abstract to provide a clearer explanation of the main findings and conclusions of the research, as per the reviewer's suggestion.

**2. The study is limited to the province of Hunan in China. In what ways, in your opinion, does the geographic focus impact the application of your findings to other regions or countries?**

**Response:** Thank you for providing the expert feedback on the geographic focus of our study. We acknowledge the importance of considering how the specific focus on Hunan Province in China may influence the generalizability of our findings to other regions or countries.

Hunan Province, situated in the heart of China, boasts a diverse and intricate topography characterized by hilly terrain and basin-like features, surrounded by three sides of land. The majority of elevations in the province are below 500 meters above sea level, with only select mountain ranges exceeding this threshold. This varied terrain presents a unique setting for studying traffic patterns and accident risks.

The varied terrain conditions in Hunan Province, including hilly areas and basin-like terrains, offer valuable insights into the factors influencing traffic accidents in similar geographic regions. We believe that the findings from our study can be extrapolated to other inland cities in China and potentially to other regions worldwide with comparable topographical and traffic characteristics.

We have carefully considered your feedback and have made amendments to our manuscript to emphasize the implications of our research findings beyond Hunan Province. Thank you for your valuable input, which has enriched the discussion on the generalizability of our study results.

**3. This study utilized traffic accident data collected by the Hunan Provincial Public Security Department in China, covering the 3-year period from 2014 to 2016. In my opinion, the data on this topic is unhelpful because it is outdated. What are the advantages of using it? but Figure 1 shows up to 2021??**

**Response:** We appreciate the thorough review of our article and the valuable feedback provided on the utilization of traffic accident data in our study. Regarding the data sourced from the Hunan Provincial Public Security Department covering the period from 2014 to 2016, we understand your concern about its potential outdatedness. However, we would like to address the advantages of using this data in our research.

Firstly, it is worth noting that the data utilized falls within the last decade, which is a common practice in traffic accident research. Numerous studies, such as those conducted by Mohan D et al. in 2017 and Wang F in 2023, have successfully used past traffic accident data to derive meaningful insights. Additionally, obtaining and utilizing traffic accident data in China involves strict confidentiality and privacy regulations, making it challenging to access more recent data without cooperation from relevant authorities.

Secondly, the focus of our study lies in comparing the effectiveness of different models, specifically the zero-truncated negative binomial model and the negative binomial model. The timeliness of the data is not the primary consideration in this model comparison. Moreover, the characteristics of certain demographic groups, such as the elderly and young, are unlikely to change significantly over a short period, making the slightly dated data still relevant for our analysis.

Lastly, despite the lack of a decreasing trend in road accident victims up to 2021, with potential impacts from the ongoing COVID-19 pandemic, we believe that the data we have utilized retains its merit in supporting our main conclusions. We acknowledge the importance of staying informed about the latest data updates and will continue to monitor for any relevant changes.

In conclusion, while we value your input, we are confident that the data used in our study remains valuable for our research objectives. Thank you once again for your insightful comments, which have enriched the discussion surrounding our data sources and analysis methods.

References:

[1] Mohan D, Bangdiwala SI, Villaveces A. Urban Street structure and traffic safety. Journal of Safety Research. 2017;62: 63-71. doi: 10.1016/j.jsr.2017.06.003. PubMed PMID: WOS:000411543500007.

[2] Wang F, Wang RQ, Yan M, Zhao JF. Does air pollution affect traffic safety? Evidence from cities in China. Environmental Science and Pollution Research. 2023;30(38):88998-9011. doi: 10.1007/s11356-023-28560-3. PubMed PMID: WOS:001030534300016.

[3] Saha D, Dumbaugh E, Merlin LA. A conceptual framework to understand the role of built environment on traffic safety. Journal of Safety Research. 2020; 75:41-50. doi: 10.1016/j.jsr.2020.07.004. PubMed PMID: WOS:000600545200006.

**4. The figures have a very low resolution.**

**Response:** Thank you for highlighting the issue regarding the resolution of the figures in our manuscript. We have taken immediate action to address this concern by replacing the figures with updated versions that meet the standard resolution requirement of no less than 300dpi per image. It is important to note that the low resolution problem was primarily caused by the PDF generation process. We have taken steps to ensure that the figures are now available in a clearer format through the downloadable image files. Your feedback is greatly appreciated, and we have made the necessary adjustments to improve the quality of the visual content in our paper. Thank you for your understanding.

**5. Given the age-specific findings, what targeted strategies would you recommend for improving the safety of vulnerable populations like children and the elderly?**

**Response:** Thank you for your insightful question regarding age-specific strategies for enhancing the safety of vulnerable populations such as children and the elderly. We greatly appreciate your valuable input, and we have incorporated targeted recommendations for improving the transportation environment for these groups in the conclusion section of our manuscript.

In response to your thoughtful suggestions, we have made significant revisions to our previous draft. Specifically, based on the study's findings, we emphasize the need to modify the traffic environment to enhance the safety of children and the elderly on high-level roadways. Proposed strategies include increasing the presence of signage, reinforcing existing signage, and implementing traffic police patrols to alert individuals to potential hazards and deter them from accessing higher-level roads.

Furthermore, our recommendations for intersections focus on the installation of pedestrian bridges in high-traffic areas to reduce accidents, the addition of pedestrian islands or sidewalks, and the creation of more median barriers to prevent pedestrians from crossing and traversing unsafe areas. These measures aim to mitigate accidents involving young individuals and the elderly on regular road segments.

We believe that these targeted strategies will contribute to creating a safer and more accessible transportation environment for vulnerable populations. Thank you once again for your valuable insights, which have enriched the discussion on improving the safety of children and the elderly in our study.

**6. How do your findings compare to similar studies conducted in other regions or countries? Are there any significant similarities or differences that you would like to highlight?**

**Response:** Thank you for your insightful inquiry regarding the comparison of our findings with similar studies conducted in other regions or countries. We sincerely appreciate your thorough review of our paper.

In alignment with findings from other scholarly studies, our research also indicates that the elderly population exhibits a lower likelihood of being involved in accidents on specialized roadways. Similarly, our study aligns with existing literature in highlighting that the likelihood of accidents increases with the road class, a trend observed by many researchers in the field. Additionally, our comparison with other studies reveals that elderly individuals exhibit unique characteristics, such as being more susceptible to accidents between lanes due to their own behaviors, a pattern consistent with findings in other research.

However, our study diverges from some existing literature in its examination of age groups. Unlike many studies that differentiate between older and younger age groups, our research reveals unexpected outcomes. Specifically, we found that adults and elderly individuals in areas with efficient traffic control measures are paradoxically more prone to traffic accidents. This unexpected result may be attributed to varying perceptions of traffic safety among individuals, with poor traffic awareness potentially contributing to this phenomenon.

We have carefully revised Section 5.2 of our manuscript to address the differences identified in comparison to other studies. These modifications aim to enhance the clarity and coherence of our research findings in relation to existing literature. Once again, we appreciate your valuable feedback, which has prompted us to refine and strengthen the discussion of our study's outcomes.

**7. Limitations and Future Research. Does this have to be in your article? It's common knowledge that no study can account for every factor.**

**Response:** Thank you for your feedback on the inclusion of limitations and future research in our article. We acknowledge the importance of recognizing the constraints and deficiencies inherent in our study, and we are fully cognizant of these limitations. By explicitly addressing these limitations and shortcomings, we aim to contribute to the advancement of future research endeavors and offer valuable insights for studies seeking to explore similar issues.

We have carefully considered your input and have revised the relevant sections of our manuscript to provide a comprehensive discussion of the study's limitations and suggestions for future research directions. We believe that this transparency will enhance the scholarly value of our work and guide the development of subsequent studies in this area. Thank you for prompting us to refine and strengthen this aspect of our paper.

**Reviewer 2**

**1. The abstract needs to include a few noteworthy findings.**

**Response:** Thank you for your feedback regarding the inclusion of noteworthy findings in the abstract. We appreciate your suggestion and have revised this section accordingly in response to your guidance. The abstract now highlights key findings of our study. We have made the necessary modifications in the manuscript to enhance the clarity and relevance of the abstract. Thank you for your valuable input, which has helped us improve the presentation of our research in the abstract section.

**2. need to arrange all figures and tables.**

**Response:** Thank you for your feedback regarding the arrangement of figures and tables in our manuscript. We have addressed this issue by uploading each figure separately and including a list of captions for both the figures and tables. This organization will enhance the clarity and accessibility of the visual content in our paper. We appreciate your guidance, and we have implemented these changes in the revised version of the manuscript to improve the overall presentation. Thank you for your valuable input.

**3. The study area map needs to add the north arrow and coordination if available. And important to add the legend to define the location of accidents.**

**Response:** Thank you for your valuable feedback regarding the study area map in our manuscript. We have carefully considered your suggestion and have made the necessary revisions to enhance the clarity and comprehensiveness of the map. Specifically, we have included a north arrow to indicate the orientation. Additionally, we have incorporated a legend in the heatmap to clearly define the locations of accidents. These enhancements will provide readers with a better understanding of the study area and the distribution of accidents. We appreciate your input, and we have implemented these changes in the revised version of the manuscript. Thank you for guiding us in improving the visual representation of our research.

**4. The regression model developed need validation or verification with field data.**

**Response:** Thank you for your insightful feedback on the validation of the regression model developed in our study. We appreciate your suggestion and have taken steps to address this concern. The zero truncated negative binomial model utilized in our research has been tested and validated in the field, as referenced in the work of Chen Y et al. This validation process has provided confidence in the reliability and effectiveness of the model for analyzing the characteristics of the elderly and young groups in our study. We have incorporated this information in the revised manuscript to emphasize the robustness of the regression model. Thank you for prompting us to clarify this aspect of our research.

References:

[4] Chen Y, Huang ZX. Predicting Wet-Road Crashes Using the Finite-Mixture Zero-Truncated Negative Binomial Model. Journal of Advanced Transportation. 2020;2020. doi: 10.1155/2020/8828939. PubMed PMID: WOS:000591563400001.

**5. What are the main recommendation that the author can advise for alleviate the accidents and reduce risk to pedestrian through utilizing transportation system in general**

**Response:** Thank you for your valuable input on recommendations for alleviating accidents and reducing risks to pedestrians through the utilization of the transportation system. We appreciate your suggestion and have incorporated specific recommendations for enhancing the transportation environment to benefit both the elderly and young populations in the conclusion section of our manuscript.

Our proposed strategy focuses on modifying the traffic environment to enhance the safety of youngsters and the elderly on high-level roadways. The study findings suggest that increasing the number of signs, reinforcing existing signage, or implementing traffic police patrols to alert individuals to potential hazards and deter them from accessing higher-level roadways are effective measures. Additionally, our recommendations for intersections include the installation of pedestrian bridges in high-traffic areas, the addition of pedestrian islands or sidewalks, and the creation of more median barriers to prevent pedestrians from crossing unsafe areas. These interventions aim to reduce accidents involving young people and the elderly on regular road segments.

We have carefully integrated these recommendations into the revised manuscript to provide practical solutions for improving pedestrian safety within the transportation system. Thank you for prompting us to enhance the discussion of recommendations in our research.

**6. All figure are not clear**

**Response:** Thank you for highlighting the issue regarding the resolution of the figures in our manuscript. We have taken immediate action to address this concern by replacing the figures with updated versions that meet the standard resolution requirement of no less than 300dpi per image. It is important to note that the low resolution problem was primarily caused by the PDF generation process. We have taken steps to ensure that the figures are now available in a clearer format through the downloadable image files. Your feedback is greatly appreciated, and we have made the necessary adjustments to improve the quality of the visual content in our paper. Thank you for your understanding.

**7. All tables are not clear.**

**Response:** Thank you for your feedback regarding the clarity of the tables in our manuscript. We have carefully reviewed and revised all tables to ensure that they are presented in a clear and easily understandable format. The content and layout of the tables have been adjusted to improve readability and enhance the presentation of data. These modifications have been implemented in the updated version of the manuscript to address the issue raised. We appreciate your attention to this matter and have taken steps to enhance the clarity of the tables in our research. Thank you for guiding us in improving the presentation of our data.

**Reviewer 3**

**1. Since the different safety effects of the various user groups were found, has the effect of the nature of pedestrians been studied in terms of the nature of physical activity and structure of the city referred to, and the extent to which this affects the analysis. The study city is considered one of the active locations in terms of business and the nature of its population.**

**Response:** Thank you for your insightful comment on the potential impact of the nature of pedestrians in relation to physical activity and the urban structure of the city on the safety effects observed in our study. We appreciate your suggestion and acknowledge the importance of considering these factors in the analysis of pedestrian safety.

In our current research, we did not specifically examine the classification of pedestrian body structure in relation to urban structure. Instead, we focused on analyzing pedestrian traffic accident data across the entire province to identify patterns and characteristics of the environments where accidents involving the elderly and young groups occurred. By identifying commonalities in these accidents, we were able to propose recommendations for improving the safety of the environment.

We recognize the significance of exploring the influence of pedestrian characteristics and urban structure on pedestrian safety outcomes, and we will take your suggestion into consideration for future studies. Your feedback is greatly appreciated, and we will strive to incorporate this aspect into our research to enhance the depth and breadth of our analysis. Thank you for your valuable input.

2. Possibility of adding more keywords.

**Response:** Thank you for your suggestion regarding the addition of more keywords to our manuscript. We appreciate your feedback and have made the necessary revisions to include "zero truncated data" and "zero truncated negative binomial models" as additional keywords in the manuscript. These keywords will help improve the visibility and searchability of our research within the academic community. We have updated the keyword section accordingly in the revised version of the manuscript. Thank you for guiding us in enhancing the keyword selection for our study.

3. The installed figures are not clear. Please install the figures in higher resolution as in figure 1.

**Response:** Thank you for highlighting the issue regarding the resolution of the figures in our manuscript. We have taken immediate action to address this concern by replacing the figures with updated versions that meet the standard resolution requirement of no less than 300dpi per image. It is important to note that the low resolution problem was primarily caused by the PDF generation process. We have taken steps to ensure that the figures are now available in a clearer format through the downloadable image files. Your feedback is greatly appreciated, and we have made the necessary adjustments to improve the quality of the visual content in our paper. Thank you for your understanding.

**Reviewer 4**

**1. The author selected traffic accident data collected by the Hunan Provincial Public Security Department in China from 2014 to 2016. The data seems out of date, and the author needs to add data from recent years (2017-2024) to derive more reliable results.**

**Response:** Thank you for your comprehensive review of our article and the insightful feedback provided on the utilization of traffic accident data in our study. We appreciate your suggestion to include more recent data from 2017 to 2024 to enhance the reliability of our results. In response to your recommendation, we have carefully considered the implications of updating the dataset and have made the following points:

The data sourced from the Hunan Provincial Public Security Department for the years 2014 to 2016, although not the most current, falls within a reasonable timeframe commonly used in traffic accident research. Past studies have successfully utilized similar historical data to derive meaningful insights, as evidenced by research conducted by Mohan D et al. in 2017 and Wang F in 2023.

Accessing and using up-to-date traffic accident data in China can be challenging due to strict confidentiality and privacy regulations. Obtaining cooperation from relevant authorities for more recent data may pose logistical difficulties. Additionally, the focus of our study on model comparison, specifically the zero-truncated negative binomial model and the negative binomial model, does not necessarily require the most current data.

The characteristics of demographic groups, such as the elderly and young, are unlikely to undergo significant changes over a short period, making the slightly dated data still relevant for our analysis. Despite the absence of a decreasing trend in road accident victims up to 2021, potentially influenced by the COVID-19 pandemic, the data we have used remains pertinent to our main conclusions.

In conclusion, while we acknowledge the importance of considering recent data updates, we believe that the data utilized in our study retains its value in supporting our research objectives. We are grateful for your valuable insights, which have enriched the discussion on our data sources and analysis methods. Thank you for your continued support and guidance in improving the quality of our research.

References:

[1] Mohan D, Bangdiwala SI, Villaveces A. Urban street structure and traffic safety. Journal of Safety Research. 2017;62:63-71. doi: 10.1016/j.jsr.2017.06.003. PubMed PMID: WOS:000411543500007.

[2] Wang F, Wang RQ, Yan M, Zhao JF. Does air pollution affect traffic safety? Evidence from cities in China. Environmental Science and Pollution Research. 2023;30(38):88998-9011. doi: 10.1007/s11356-023-28560-3. PubMed PMID: WOS:001030534300016.

[3] Saha D, Dumbaugh E, Merlin LA. A conceptual framework to understand the role of built environment on traffic safety. Journal of Safety Research. 2020;75:41-50. doi: 10.1016/j.jsr.2020.07.004. PubMed PMID: WOS:000600545200006.

**2. Did the author only consider collisions? What about other accidents, such as fire, explosion, scraping and rolling etc.?**

**Response:** Thank you for your valuable feedback on our study. We appreciate your question regarding whether we only considered collisions or other types of accidents, such as fire, explosion, scraping, and rolling. In our research, we specifically focused on analyzing pedestrian-vehicle collisions from the perspective of pedestrian travel. We believe that pedestrian-vehicle collisions are a prevalent and representative form of traffic accidents that warrant detailed study.

While our current study concentrated on pedestrian-vehicle collisions, we acknowledge the importance of considering a broader range of accident types in future research. Your suggestions regarding other accident scenarios, including fire, explosion, scraping, and rolling, are insightful and provide valuable directions for expanding the scope of our analysis. We will take your recommendations into account and explore the incorporation of these factors in our future research endeavors.

We have carefully considered your feedback and will strive to enhance the comprehensiveness of our research by incorporating a more diverse range of accident types in our future studies. Thank you for your constructive input, which has prompted us to further develop our research methodology and broaden the scope of our analysis.

3. The resolution of the figures is too low, and it is difficult for me to understand them. The author is encouraged to provide vectograph.

**Response:** Thank you for highlighting the issue regarding the resolution of the figures in our manuscript. We have taken immediate action to address this concern by replacing the figures with updated versions that meet the standard resolution requirement of no less than 300dpi per image. It is important to note that the low resolution problem was primarily caused by the PDF generation process. We have taken steps to ensure that the figures are now available in a clearer format through the downloadable image files. Your feedback is greatly appreciated, and we have made the necessary adjustments to improve the quality of the visual content in our paper. Thank you for your understanding.

4. The language of this manuscript needs to be polished by a native speaker.

**Response:** Thank you for your feedback on the language quality of our manuscript. We have taken your suggestion seriously and have engaged the services of a native speaker to help polish the language in our paper. The revised version of the manuscript has undergone thorough language editing to ensure clarity, coherence, and accuracy in the presentation of our research findings. We appreciate your attention to this aspect of our work and have made the necessary improvements to enhance the overall quality of the manuscript. Thank you for guiding us in improving the language proficiency of our paper.

**5. The application value of the article should be more prominent in the Abstract part.**

**Response:** Thank you for your feedback on the prominence of the application value in the Abstract of our manuscript. We have carefully reviewed and revised the Abstract to ensure that the application value of our research is more prominently highlighted. In the revised version, we have emphasized the practical significance and potential impact of our findings on the field of transportation engineering. By clearly articulating the practical implications of our study, we aim to better communicate the value of our research to the readers. We appreciate your guidance in enhancing the Abstract section of our paper and have made the necessary improvements to underscore the application value of our work. Thank you for your valuable input.

**Reviewer 5**

**1. In fact, there are many derived models of negative binomial models, but there are few methods and models in the introduction, especially for the application and advantages of ZTNB model, the description is insufficient.**

**Response:** We sincerely appreciate your thorough review and insightful feedback on our article. We have carefully considered your suggestions and have made revisions to enhance the description of the zero truncated negative binomial (ZTNB) model in Section 3.3.1 of the manuscript.

The ZTNB model offers distinct advantages over the traditional negative binomial (NB) model by addressing the presence of only non-zero accident counts in the dataset, excluding zero values. This distinctive feature is achieved through the incorporation of an additional parameter that captures the probability of observing a non-zero outcome, leading to more precise estimates of the regression coefficients.

Estimation of the ZTNB model involves maximum likelihood techniques, with adjustments made to the log-likelihood function to accommodate the truncation at zero. The resulting coefficient estimates can be interpreted similarly to those of the NB model, reflecting the expected change in the log of the non-zero accident count associated with a unit change in the corresponding explanatory variable.

By elaborating on the unique characteristics and estimation methods of the ZTNB model, we aim to provide a clearer understanding of its application and advantages in our research. Your feedback has been invaluable in guiding us to enhance the description of the ZTNB model in our manuscript. Thank you for your expertise and constructive input, which have contributed to the refinement of our study.

**2. The author selected traffic accident data collected by the Hunan Provincial Public Security Department in China from 2014 to 2016. The data seems out of date, and the author needs to add data from recent years (2017-2024) to derive more reliable results.**

**Response:** Thank you for your comprehensive review of our article and the insightful feedback provided on the utilization of traffic accident data in our study. We appreciate your suggestion to include more recent data from 2017 to 2024 to enhance the reliability of our results. In response to your recommendation, we have carefully considered the implications of updating the dataset and have made the following points:

The data sourced from the Hunan Provincial Public Security Department for the years 2014 to 2016, although not the most current, falls within a reasonable timeframe commonly used in traffic accident research. Past studies have successfully utilized similar historical data to derive meaningful insights, as evidenced by research conducted by Mohan D et al. in 2017 and Wang F in 2023.

Accessing and using up-to-date traffic accident data in China can be challenging due to strict confidentiality and privacy regulations. Obtaining cooperation from relevant authorities for more recent data may pose logistical difficulties. Additionally, the focus of our study on model comparison, specifically the zero-truncated negative binomial model and the negative binomial model, does not necessarily require the most current data.

The characteristics of demographic groups, such as the elderly and young, are unlikely to undergo significant changes over a short period, making the slightly dated data still relevant for our analysis. Despite the absence of a decreasing trend in road accident victims up to 2021, potentially influenced by the COVID-19 pandemic, the data we have used remains pertinent to our main conclusions.

In conclusion, while we acknowledge the importance of considering recent data updates, we believe that the data utilized in our study retains its value in supporting our research objectives. We are grateful for your valuable insights, which have enriched the discussion on our data sources and analysis methods. Thank you for your continued support and guidance in improving the quality of our research.

References:

[1] Mohan D, Bangdiwala SI, Villaveces A. Urban street structure and traffic safety. Journal of Safety Research. 2017;62:63-71. doi: 10.1016/j.jsr.2017.06.003. PubMed PMID: WOS:000411543500007.

[2] Wang F, Wang RQ, Yan M, Zhao JF. Does air pollution affect traffic safety? Evidence from cities in China. Environmental Science and Pollution Research. 2023;30(38):88998-9011. doi: 10.1007/s11356-023-28560-3. PubMed PMID: WOS:001030534300016.

[3] Saha D, Dumbaugh E, Merlin LA. A conceptual framework to understand the role of built environment on traffic safety. Journal of Safety Research. 2020;75:41-50. doi: 10.1016/j.jsr.2020.07.004. PubMed PMID: WOS:000600545200006.

**3. It is suggested that the authors provide a more detailed explanation of the selection of road environment variables, particularly explaining why certain variables were included in the analysis while other potentially important factors (e.g., weather conditions, time factors) were not considered, to ensure the comprehensiveness of the research results.**

**Response:** Thank you for your valuable feedback and suggestion. We appreciate your insight into the need for a more detailed explanation of the selection of road environment variables in our study. We acknowledge the importance of ensuring the comprehensiveness of our research results and will address this in the revised manuscript.

In our research, we focused on examining the relationship between the number of accidents and the road environment. When selecting road sections for analysis, we collected statistical data on various environmental variables along with accident counts. While we understand the significance of factors such as weather conditions and time of day in influencing road safety, these variables were not included in our analysis as they are considered external factors that are not directly controlled by road environment conditions.

Our objective was to investigate the impact of road infrastructure and design on accident occurrence by examining factors such as roadway facilities, road type, and other related variables. By studying the relationship between traffic environment characteristics and accident frequency, we aim to identify areas where improvements can be made to enhance road safety outcomes within our control.

We will provide a more detailed explanation in the manuscript regarding the rationale behind the selection of road environment variables and the exclusion of certain external factors. Your feedback has prompted us to clarify and elaborate on our research methodology to ensure transparency and thoroughness in our analysis. Thank you for guiding us in improving the clarity and comprehensiveness of our study.

**4. It is recommended to further strengthen the policy recommendations in the conclusion section, clearly specifying which road environment improvement measures are most likely to effectively reduce traffic accidents, and providing specific implementation paths. This will offer more actionable advice for policymakers and urban planners.**

**Response:** Thank you for your recommendation. We have further strengthened the policy recommendations in the conclusion section of the manuscript, providing specific suggestions for improving the road environment to effectively reduce traffic accidents, particularly focusing on enhancing safety for vulnerable demographic groups such as the elderly and young individuals.

Our proposed strategies include the following: The study findings indicate the need to modify the traffic environment to better protect adolescents and seniors on higher-grade roads. Methods to achieve this goal may involve increasing the number of road signs, reinforcing existing signage, or establishing traffic patrol teams to alert individuals to potential dangers and deter them from accessing higher-grade roads. In busy areas, our recommendations for intersections include installing pedestrian overpasses to reduce accident rates, adding pedestrian overpasses or sidewalks, and increasing the number of median barriers to prevent individuals from crossing from safe areas. These measures aim to prevent accidents involving young and elderly individuals on regular road segments.

By offering these specific suggestions for enhancing the road environment and safety measures, we aim to provide actionable advice for policymakers and urban planners. These recommendations are intended to guide the implementation of effective measures to reduce traffic accidents and safeguard vulnerable road users. Thank you for guiding us to provide more detailed and practical policy recommendations in our study.

**5. In the fourth paragraph of Section 5.3.1, there are repeated statements regarding the quantity of each sample.**

**Response:** Thank you for your feedback. We have carefully reviewed the fourth paragraph of Section 5.3.1 and have addressed the issue of repeated statements regarding the quantity of each sample in the revised manuscript. We have revised the paragraph to eliminate redundancy and ensure clarity and conciseness in our presentation of the sample sizes. The modifications made aim to enhance the coherence and readability of the section, providing a more streamlined and focused discussion of the sample quantities. Thank you for bringing this to our attention, and we appreciate the opportunity to refine and improve the clarity of our research findings.
