# Supplementary material for: Influence of road environmental factors on traffic accidents involving vulnerable road users through negative binomial models
Source: PLoS One. 2025 Feb 13;20(2):e0317601. doi: 10.1371/journal.pone.0317601 (PMC11824970; doi:10.1371/journal.pone.0317601)
Supplement: Attachment — Submitted filename: Response to Reviewers.docx [file pone.0317601.s005.docx]

**Author's Response to Reviewer’s Comments**

Dear Editors and Reviewers:

Thank you for your efficient work in processing our manuscript (PONE-D-24-31325). Those comments are very beneficial to improve our paper, and they also can be seen as the important guidance to our research. We have studied comments carefully and have made targeted revisions. The changes are marked red in the revised manuscript. Point by point responses to the reviewers’ comments are listed below.

**Reviewer 2**

**1.** **Need to add only limitation for the work more clearly, and all the required notes has been done. Also, and all the questions has been clarified.**

**Response:** Thank you for your careful reading and guidance. We have added information about the limitations of our work at the end. We have added the following words to the **Limitations and Future Research** section of the text.

Due to some legal and regulatory restrictions, accident data for nearby years cannot be obtained. This does not prevent us from identifying some accident characteristics of the elderly and young population from accident data over the past 10 years, but we still hope to obtain the latest data to make our research more comprehensive.

**Reviewer 3**

**1. The researcher made all the modifications required by him and determined by the four evaluators.**

**Response:** Thank you for your careful reading and suggestions We will remember them and continue to improve them in our future work.

For each key comment, I provided in-depth analyses. For example, in response to queries about research methodology, I provided a more detailed description of the experimental design and introduced additional data to validate the reliability and stability of the findings; for suggestions about the theoretical framework or background of the literature, I expanded the literature review section and further articulated how my study fills a gap in the current field or deepens existing understanding.

**2. The researcher answered most of the research inquiries directed by reviewers.**

**Response:** Thank you for recognising my research work and for your detailed review of the paper. It is a great relief to know that I have answered most of the research questions raised by the reviewers. During the revision process, I carefully considered each review comment and endeavoured to answer these questions through logical and clear responses, refinement of data analysis, and content additions.

While I have endeavoured to answer all questions, I also recognise that there may still be room for further improvement in some areas. I will continue to pay attention to these details and improve them in future research.

Thank you again for your support of my work. If there is anything else that needs to be further refined or added, I am more than willing to make any adjustments or improvements.

**3. Although the study period was proposed, which is the years 2014 to 2016, and although the years are not specific, the analysis was done in good ways and the study can be used for good future research.**

**Response:** Thank you for recognising my research and the value of the thesis! It is a great pleasure to learn that you found my research methodology analytically sound and that the results can provide a good reference point for future research. Your encouragement means a lot to me and it motivates me to continue exploring this field in the future.

Regarding your reference to the timeframe of the study (2014 to 2016), while the selection of these years may not have been explicitly specified in detail, I did attempt to compensate for this potential limitation in the study in a reasonable manner. For example, I combined a variety of statistical and modelling methods during the data cleaning and analysis phase to ensure that the study's findings were scientifically sound and representative. Also, in the discussion section, I explicitly emphasised the applicability and limitations of the study's timeframe and made suggestions that future studies could extend the time span or incorporate additional time periods to validate the findings.

I would also like to add that although the time span of the study is relatively short, I chose this range based on the completeness of the data at the time and its’ fit with the context of the study and it provides a baseline reference for future long-term analyses.

Thank you again for recognising the potential academic contribution of this paper! Your feedback has made me acutely aware of the room for improvement in research design and presentation. In the follow-up study, I will pay more attention to refining the logic of choosing the time of the study, as well as adding more detailed descriptions as much as possible to enhance the persuasiveness and generalisability of the study.

**Reviewer 5**

**1. The reviewer recommends acceptance for publication.
The author has responded to the reviewers' comments and made corresponding revisions according to their suggestions. There are no further issues.**

**Response:** I would like to thank the reviewers for recognising my paper and recommending it for publication! Thank you very much for your valuable comments and detailed feedback throughout the review process, which not only played an important role in improving the quality of the paper, but also benefited me in my research and writing.

During the revision process, I have endeavoured to refine the detailed content of the article and address all the suggestions. I am honoured to learn that there are no further issues with the thesis for this time! If there are any other subsequent additions that need to be made, I will continue to work on them and improve them further.

Thank you again for your time and for recognising my work!
